# Differential diagnosis between benign and malignant pleural effusion with dual-energy spectral CT

**DOI:** 10.1371/journal.pone.0193714

**Published:** 2018-04-11

**Authors:** Xirong Zhang, Haifeng Duan, Yong Yu, Chunling Ma, Zhanli Ren, Yuxin Lei, Taiping He, Ming Zhang

**Affiliations:** 1 Department of Radiology, the First Affiliated Hospital of Xi’An Jiaotong University College of Medicine, Xi’An city, Shaanxi Prov, China; 2 Department of Radiology, Affiliated Hospital of Shaanxi University of Chinese medicine, Xianyang city, Shaanxi Prov, China; 3 Department of Medical Techniques, Shaanxi University of Chinese medicine, Xianyang city, Shaanxi Prov, China; Universitair Medisch Centrum Utrecht, NETHERLANDS

## Abstract

**Purpose:**

To investigate the value of spectral CT in the differential diagnosis of benign from malignant pleural effusion.

**Method and materials:**

14 patients with benign pleural effusion and 15 patients with malignant pleural effusion underwent non-contrast spectral CT imaging. These patients were later verified by the combination of disease history, clinical signs and other information with the consensus of surgeons and radiologists. Various Spectral CT image parameters measured for the effusion were as follows: CT numbers of the polychromatic 140kVp images, monochromatic images at 40keV and 100keV, the material density contents from the water, fat and blood-based material decomposition images, the effective atomic number and the spectral curve slope. These values were statistically compared with t test and logistic regression analysis between benign and malignant pleural effusion.

**Results:**

The CT value of benign and malignant pleural effusion in the polychromatic 140kVp images showed no differences (12.61±3.39HU vs. 14.71±5.03HU) (P>0.05), however, they were statistically different on the monochromatic images at 40keV (43.15±3.79 vs. 39.42±2.60, p = 0.005) and 100keV (9.11±1.38 vs. 6.52±2.04, p<0.001). There was difference in the effective atomic number value between the benign (7.87±0.08) and malignant pleural effusion (7.90±0.02) (P = 0.02). Using 6.32HU as the threshold for CT value measurement at 100keV, one could obtain sensitivity of 100% and specificity of 66.7% with area-under-curve of 0.843 for differentiating benign from malignant effusion. In addition, age and disease history were potential confounding factors for differentiating malignant pleural effusion from benign, since the older age (61.13±12.51 year-old vs48.57±12.33 year-old) as well as longer disease history (70.00±49.28 day vs.28.36±21.64 day) were more easily to be found in the malignant pleural effusion group than those in the benign pleural effusion group. By combining above five factors, one could obtain sensitivity of 100% and specificity of 71.4% with area-under-curve of 0.933 for differentiating benign from malignant effusion.

**Conclusion:**

The CT value measurement at both high and low energy levels and the effective atomic number obtained in a single spectral CT scan can assist the differential diagnosis of benign from malignant pleural effusion.Combining them with patient age and disease history can further improve diagnostic performance.

**Clinical relevance/Application:**

Clinical findings and Spectral CT imaging can provide significant evidences about the nature of pleural effusion.

## Introduction

Pleural effusion is a condition where there is a buildup of abnormal fluid within the pleural space, its causes include cardiac insufficiency, malignant tumor, tuberculous pleuritis, pulmonary embolism, pneumonia and so on. According to the characteristics of pleural effusion classification, it can be divided into leakage, exudate (serous or bloody), empyema, hemothorax, chylothorax. One of the major issues in the differential diagnosis of pleural effusion is early recognition and differentiating benign from malignant pleural effusion. Early treatment of benign pleural effusion is curative and decreases the possibility of complications, meanwhile early treatment of malignant pleural effusion may increase quality of life and survival of patients with advanced malignant disease[1–3].

However, the differential diagnosis of pleural effusion sometimes represents a considerable challenge. Because the conventional methods, such as the direct examination of pleural fluid by Ziehl-Neelsen staining, culture of the pleural fluid, and blind pleural biopsy[4, 5] are not always helpful, about 20–40% of patients with pleural effusion remain undiagnosed[2], and more invasive procedures like medical thoracoscopy (MT)[2, 6, 7] or thoracotomy are needed to perform differential diagnosis. Meanwhile, we must keep in mind that MT remains an invasive procedure requiring training and careful patient selection, and the patients who have pleural adhesion is not recommended for MT.

Diagnosis of pleural effusion in radiology, such as chest CT examination determines the severity of pleural effusion[8]. However, conventional CT is unable to determine whether pleural effusion is benign or malignant, and additional tests are often required. In recent years, there has been an increasing interest in the use of dual-energy spectral CT imaging for the evaluation of pleural effusion, which can generate material decomposition images as well as monochromatic image sets with fast kilovoltage switching[9–11]. At present, the study of chest dual energy spectral CT, mainly concentrated in the low dose and contrast agent, differential diagnosis of benign and malignant pulmonary nodules[12], but there are few studies on pleural effusion. The aim of this study was to investigate the value of spectral CT in the differential diagnosis of benign from malignant pleural effusion.

## Materials and methods

### Patient information

In this retrospective study, all research procedures were approved by the Ethics Committee of Affiliated Hospital of the Shaanxi University of Chinese Medicine.Written informed consent was obtained from all patients, or their legal representatives, prior to inclusion. From August 2013 to February 2014, fifty-five patients who suffered from pleural effusion and scanned with dual-energy spectral CT were collected. Exclusion criteria were: (1) the reason for pleural effusion was not clear, (2) pleural effusion was too small to affect the measurement results, (3) patients who undergone surgical treatment with pleural effusion before dual-energy spectral CT. Sixteen cases were excluded (n = 5 for the not clear cause for pleural effusion, n = 5 for small pleural effusion and n = 6 for under surgical treatment). Finally, twenty-nine patients who suffered from pleural effusion were included. The diagnoses were confirmed by the combination of patient history, clinical signs and other information with the consensus of surgeons and radiologists. The diagnostic methods for determining the nature of pleural effusion included 13 patients with clinical findings, 4 with thoracotomy, 2 with thoracoscope, 2 with bronchoscopy and 8 with thoracic puncture.

Patients were divided into two groups for retrospective analysis: Group I of 14 patients with benign pleural effusion (8 with pneumonia, 5 with pulmonary tuberculosis and 1 with pneumocystosis) and Group II of 15 patients with malignant pleural effusion (5 with adenocarcinoma of lung, 4 with squamous cell carcinoma of lung, 2 with adenosquamous carcinoma of lung and 4 with transitivity carcinoma of lung).

### Scan technique

All patients were scanned on a 64-section CT scanner (Discovery CT750 HD; GE Healthcare) using the helical spectral CT imaging mode with fast tube voltage switching between 80kVp and 140kVp in less than 0.5ms during gantry rotation. All patients were scanned feet-first in supine position. The scan range was from the top of the lungs to the top of the diaphragm. Other scanning parameters were the following: rotation speed, 0.6 s/rot; tube current, 600mA; scan slice thickness,5mm; reconstruction slice thickness, 0.625mm; helical pitch, 0.984; collimation, 40mm. Before examination, patients underwent respiratory exercise by technicians to reduce the artifacts caused by respiratory motion.

### Image processing and data measurement

The material decomposition images and monochromatic images with the slice thickness of 0.625 mm and spacing of 0.625 mm were reconstructed and sent to an advanced workstation for image analysis and data measurement using the Gemstone Spectral Imaging (GSI) viewer (GSI viewer 2.00 and GE Volume Share 4 AW 4.4, GE Healthcare). Specifically, the following image sets were used for analysis: image sets at polychromatic 140kVp, monochromatic 40keV and 100keV energy levels for CT value measurement; water, fat and blood-based material decomposition images were extracted for water, fat and blood density measurement, respectively. Spectral HU curve (CT value measurement as function of photon energy in spectral CT) was generated to calculate the effective atomic number and the spectral curve slope (λ).

**Fig 1:**
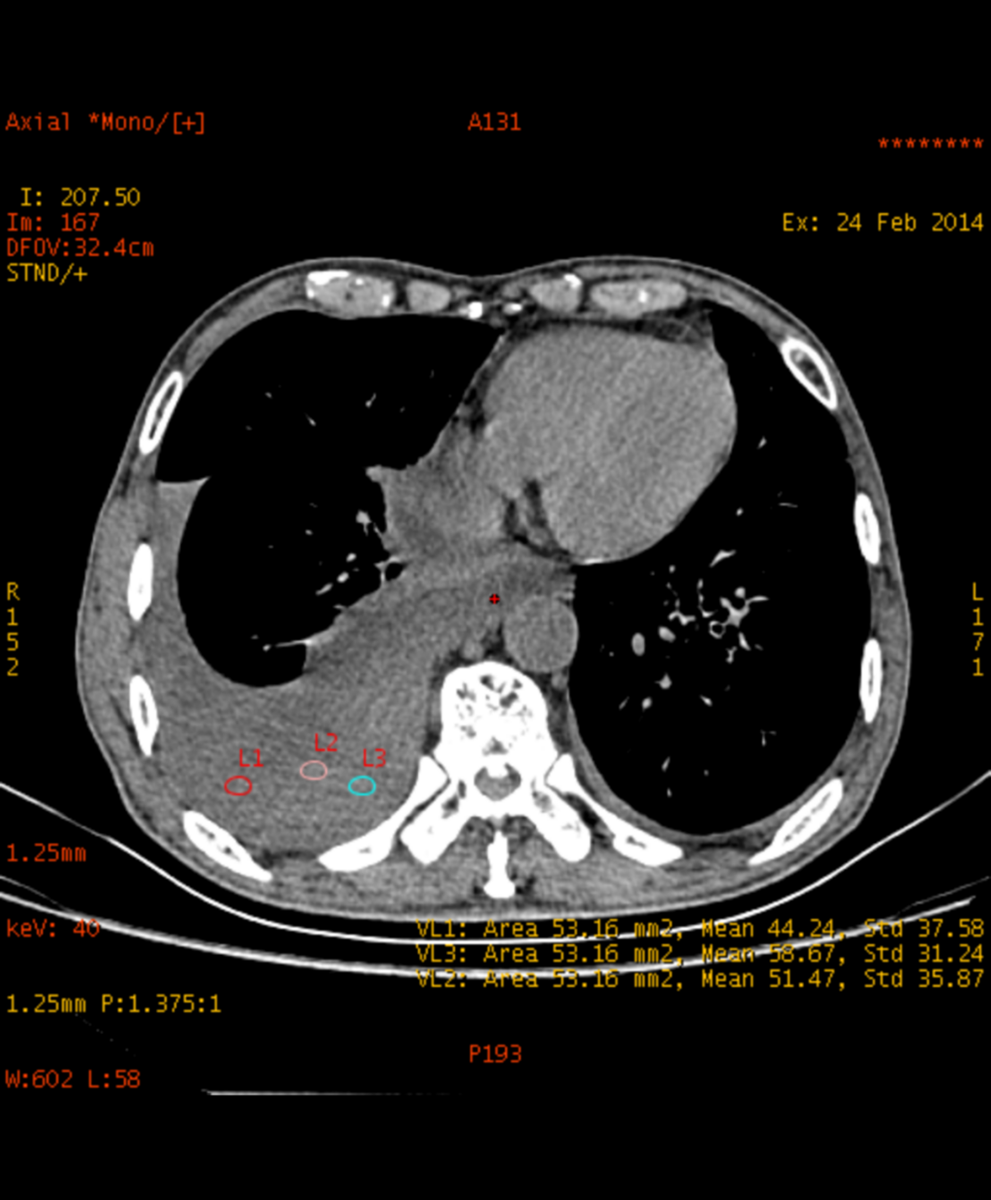
ROI size and location in the monochromatic images at 40keV.

Because different series could not be loaded simultaneously, three regions of interest (ROIs) (the relative homogeneous area) were used on the non-enhanced images. The size and shape of ROIs were the same by using the copy-and-paste function in every patient. The patients included in this study suffered from pleural effusion with different amount, so the area of the ROIs ranging from 40.86 mm2 to 70.66 mm2 was positioned in the center area of the pleural effusion and keep away from artifacts **(Fig 1**). For the objective measurement, a radiologist (Y.Y.) with 10-years of experience in chest CT determined ROI size and location. Measurements were repeated three times within the pleural effusion for each patient and averaged to produce the final results.

### Statistical analysis

Statistical analysis was performed using commercially available software (version 17.0 for Windows, SPSS Inc.). Data were expressed as mean ± standard deviations. The multiple logistic regression analysis was used to compare the various patients’ characteristics between benign and malignant pleural effusion. The single-sample K-S analysis was used to test the homogeneity of variance of the measurements. The independent samples student-t test was used to test the CT parameters when data were consistent with normal distribution, including CT numbers of the polychromatic 140kVp images, monochromatic images at 40keV and 100keV, the material density contents from the fat and blood-based material, the effective atomic number and the spectral curve slope. Otherwise the Mann-Whitney U test was used to test the CT parameters (the material density contents from the water-based material decomposition images). Candidate factors found to have a p-value< 0.05 in the initial analyses were included in receiver operating characteristic (ROC) analysis. The ROC curves were generated to establish the optimal threshold values to distinguish benign and malignant pleural effusion according to the maximum Youden Index (YImax = sensitivity+specificity-1), and to calculate the sensitivity and specificity. The diagnostic capability was determined by calculating the area under the ROC curve (AUC).

## Results

### Comparison of patients’ characteristics between benign and malignant pleural effusion

The patients in our study included 22 males and 7 females, and ages ranged from 35 to 86 years with mean age of 55.8 years. Group I included 11 males and 3 females, and ages ranged from 35 to 73 years with mean age of 48.57 years. Group II included 11 males and 4 females, and ages ranged from 41 to 86 years with mean age of 61.13 years. The logistic regression analysis showed that age and disease history were the potential confounding factors to differentiating benign from malignant pleural effusion (P = 0.011 and 0.001, respectively). However, gender, and clinical symptoms (fever, cough and chest pain) were not potential confounding factors (P = 1.000, 0.085, 0.647, 0.214, respectively) (**Table 1**).

**Table 1:** Patient characteristics.

	Benign(n = 14)	Malignant(n = 15)	*p*
Age(*years*)	48.57±12.33	61.13±12.51	0.011
Gender(*F/M*)	3/11	4/11	1.000
Disease history(days)	28.36±21.64	70.00±49.28	0.001
Fever/NO fever	9/5	4/11	0.085
Cough/NO cough	7/7	9/6	0.647
Chest pain/NO chest pain	3/11	2/13	0.214

### Comparison of measurements in spectral CT between benign and malignant pleural effusion

**Fig 2:**
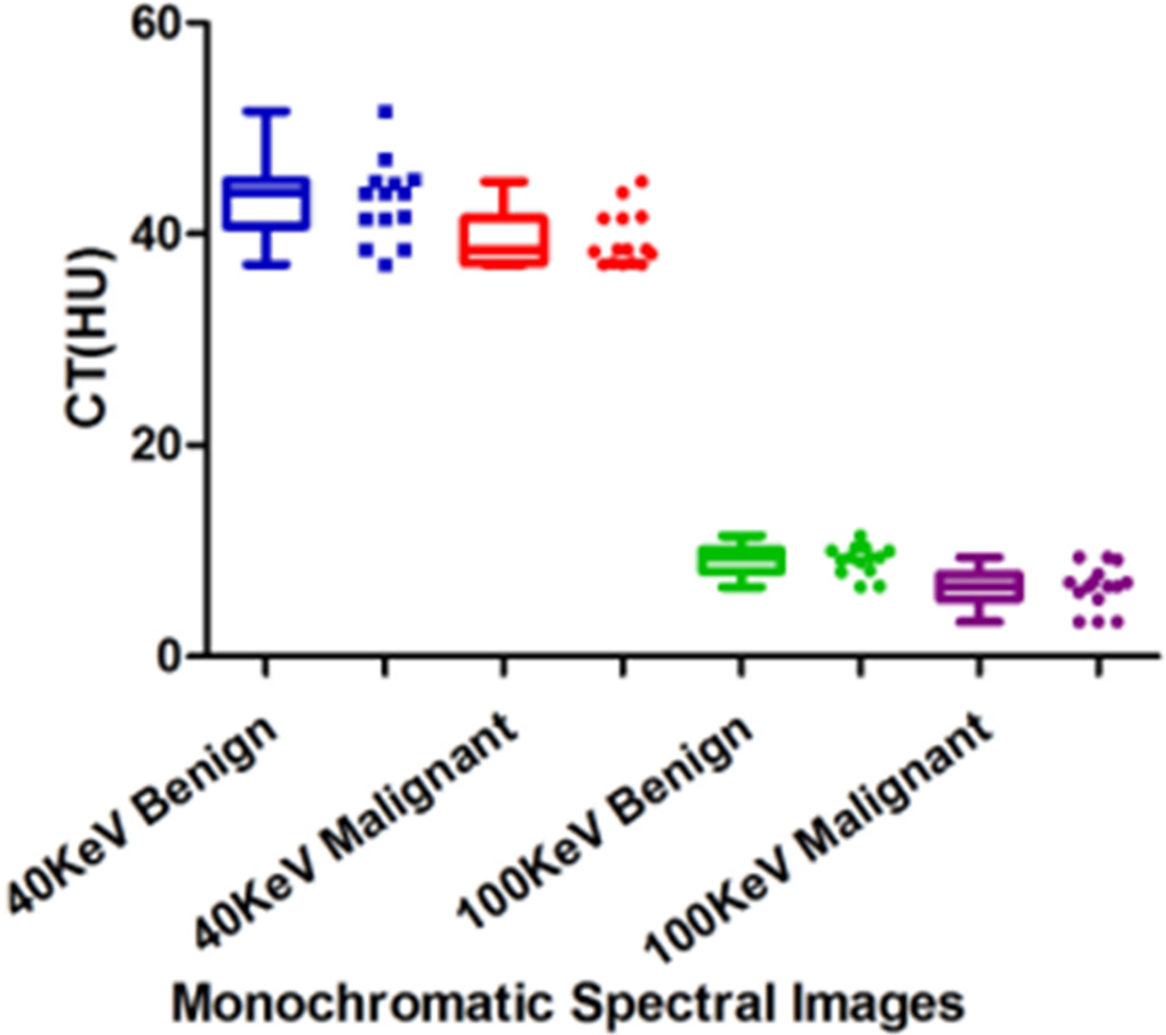
Parallel boxplots and scatter plot of monochromatic images at 40keV and 100keV.

The quantitative measurements in spectral CT imaging included CT values at different photon energy levels and material density values from different material bases. All of the CT parameters were found to have normal distribution (Skewness and Kurtosis are both less than 1). CT values between the benign and malignant pleural effusion in the conventional 140kVp images showed no difference (12.61±3.39HU vs. 14.71±5.03HU, P = 0.20). The water-density, fat-density, blood-density and the spectral curve slope measurements in the material decomposition images in spectral CT did not show any difference between the benign and malignant pleural effusion either (P>0.05). However, CT values between the benign and malignant pleural effusion were statistically different at both 40keV and 100keV monochromatic spectral images (43.15±3.79HU vs. 39.42±2.60HU at 40keV, p = 0.005; 9.11±1.38HU vs. 6.52±2.04HU at 100keV, p<0.001). The effective atomic number value of benign pleural effusion was 7.87±0.08, which was statistically different from that of the malignant pleural effusion (7.90±0.02, P = 0.02) (**Fig 2 and Table 2**).

**Table 2:** Comparison of measurements in spectral CT between benign and malignant pleural effusion.

Parameter	Benign(n = 14)	Malignant(n = 15)	*p*
CT Value (HU)			
140KVp	12.61±3.39	14.71±5.03	0.20
40keV	43.15±3.79	39.42±2.60	0.005
100keV	9.11±1.38	6.52±2.04	<0.001
Effective Atomic Number	7.87±0.08	7.90±0.02	0.02
Spectral Curve Slope	0.56±0.05	0.60±0.05	0.06
Density (mg/ml)			
Water-based	1016.87±19.84	1008.4±12.22	0.18
Fat-based	-379.7±38.68	-392.6±41.28	0.39
Blood-based	1625.3±98.44	1655.8±106.24	0.33

**Fig 3:**
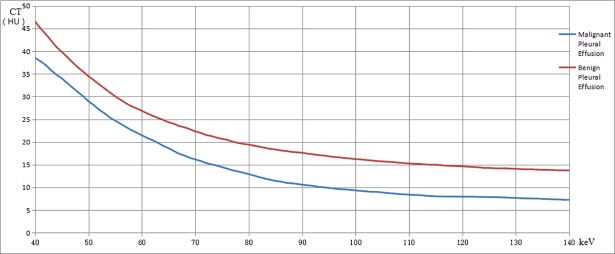
The spectral slope curve of benign pleural effusion and malignant pleural effusion.

The ROC analysis was performed using the CT value measurement and effective atomic number. Using 38.4HU, 6.32HU and 7.885 as the threshold value for CT value measurement at 40keV and 100keV and effective atomic number, respectively, we could obtain sensitivity of 92.5%, 100% and 80% and specificity of 53.5%,66.7% and 71.4% with area-under-curve (AUC) of 0.795, 0.843 and 0.740 for differentiating benign from malignant pleural effusion. In addition, our data indicated that, although the slopes of the spectral curve of benign and malignant pleural effusion showed no difference, the overall spectral curve of the benign pleural effusion was lower than the malignant (**Fig 3**).

### Measurements in spectral CT and patient characteristics between benign and malignant pleural effusion in ROC analysis

**Fig 4:**
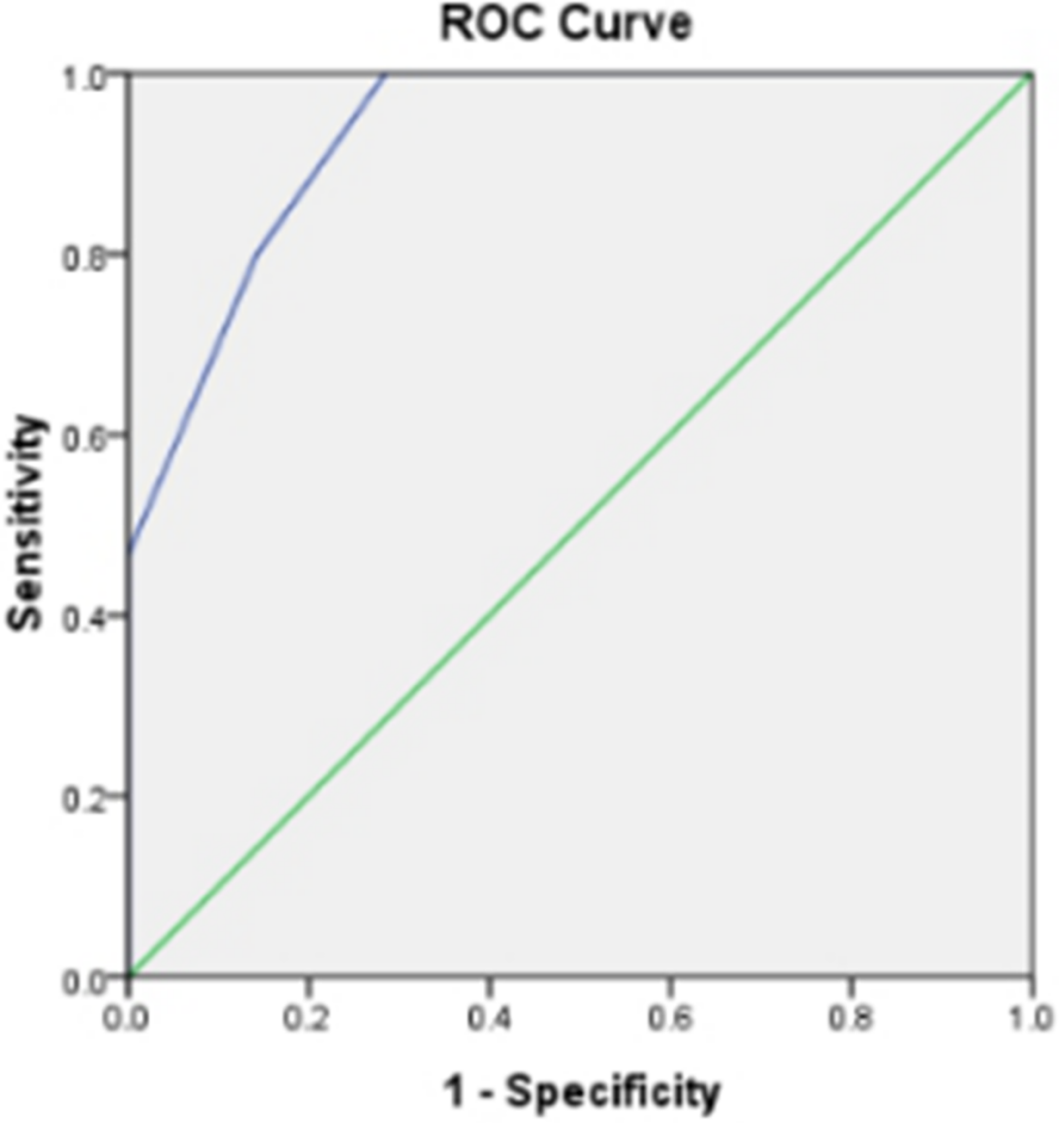
Receiver operating characteristic curve for factors (combine age, disease history, effective atomic number, monochromatic images at 40keV and 100keV) in differentiating benign pleural effusion from malignant pleural effusion (sensitivity of 100% and specificity of 71.4% with area-under-curve of 0.933).

Because there were potential confounding factors in this study, another ROC analysis was performed combining the age, disease history, effective atomic number, CT value measurement for monochromatic images at 40keV and 100keV. By combining these five factors we could obtain sensitivity of 100% and specificity of 71.4% with area-under-curve of 0.933 for differentiating benign from malignant pleural effusion **(Fig 4 and Table 3)**.

**Table 3:** The diagnostic performance of the age,disease history,CT value in 40keV, 100kev and effective atomic number to differentiate between benign and malignant pleural effusion.

	AUC	YI	Threshold	Sensitivity(%)	Specificity(%)
Age(*years*)	0.771	0.524	60.5	66.7	85.7
Disease history(days)	0.876	0.596	47.5	66.7	92.9
40 keV(HU)	0.795	0.452	38.4	92.5	53.5
100keV (HU)	0.843	0.657	6.32	100	66.7
Effective Atomic Number	0.740	0.514	7.885	80.0	71.4

AUC: Area-under-curve

YI: Youden index

## Discussion

Most of the pleural effusion can be confirmed by many clinical tools, for example biochemical or microbiological examinations, cytological analysis of pleural fluid, and biopsy of pleura. But there are a few patients in whom the cause of pleural effusion has remained unclear, especially for the malignant pleural effusion. Studies [8, 12] have indicated that regular chest CT images can be used to evaluate the severity of pleural effusion, and to detect malignant pleural effusion with high sensitivities (ranging from 88% to 100%) but low specificities (ranging from 22% to 56%) [13–15]. There have been publications focusing on differentiating pleural exudates from transudates by conventional CT[16–18]. In the study by Çullu N et al.[16], CT attenuation value is shown to be useful in differentiating exudates from transudates, exudate can be considered when the CT attenuation values are >15 HU, its close correlation with clinical findings is essential. However, few published studies have evaluated the nature of pleural effusion by dual-energy spectral CT.

In this study, we evaluated the clinical value of dual-energy spectral CT for the differential diagnosis between benign and malignant pleural effusion. Our study had shown that CT values of the benign and malignant pleural effusion were statistically different (P<0.05) at both 40keV and 100keV monochromatic spectral CT images. The effective atomic number value of benign pleural effusion was also statistically different from that of the malignant pleural effusion (P<0.05). Using CT value measurement on the monochromatic spectral CT images at 100keV, one could obtain a high sensitivity (100%) and moderate specificity (66.7%) to differentiate benign from malignant pleural effusion. Age and disease history are the potential confounding factors to differentiating benign from malignant pleural effusion, that is the age and disease history in patients with benign pleural effusion are less than in malignant patients. Because these five candidate factors are easy to obtain clinically, and were considered together in this study to identify benign and malignant pleural effusion by ROC curves. We could obtain a perfect sensitivity (100%), moderate specificity (71.4%) and high area-under-curve (0.933) for differentiating benign from malignant pleural effusion. Our study demonstrated that combined with age and disease history, the effective atomic number and CT values at the 40keV and 100keV monochromatic images obtained in the non-enhanced dual-energy spectral CT imaging can effectively distinguish the benign and malignant pleural effusion.

The obtained specificity with dual-energy spectral CT was a big improvement over the conventional CT. This is largely due to the fact that conventional CT uses polychromatic lower-energy x-ray beams that produces average attenuation across a wide energy spectrum. The average effect reduces the low contrast resolution, and makes it harder to separate materials with small density difference, such as the benign and malignant pleural effusion. Unlike conventional CT imaging, spectral CT acquisition is a novel imaging technique which generates monochromatic and material decomposition images[19]. On the other hand, using fast kilovoltage switching between high and low tube voltages (140 kVp and 80 kVp) in a single X-ray tube in less than 0.5ms coupled with a high-performance gemstone detector and powerful image post-processing [10,20], spectral CT generates both material decomposition images and a set of monochromatic images with energy levels from 40-140keV. The creation of monochromatic image sets not only improves the low contrast resolution in comparison with the polychromatic images[10], but also provides the ability to select energy levels where the two types of effusions exhibit the greatest difference in CT values.

In addition, benign and malignant pleural effusion have different cytology ingredients: malignant pleural effusion has much richer carcinoembryonic antigen, endothelial growth factors, haemocytes and hemoglobin than benign pleural effusion [21,22]. These ingredients generate different X-ray absorptions, not big enough to be detected when polychromatic x-ray beam (140kVp images) were used, but were evident in the monochromatic 40keV and 100keV spectral CT images. The effective atomic number can be calculated in spectral CT imaging to reflect the intrinsic material composition of tissue and lesion[10]. It is a unique parameter generated only in spectral CT imaging and also showed statistically significant difference between the benign and malignant pleural effusion, reflecting the intrinsically different material compositions.

Age and disease history were found to be the potential confounding factors for differentiating benign from malignant pleural effusion, these were consistent with our daily awareness. In order to get more accurate results, age and disease history as the important factors should to be taken into account in differentiating benign from malignant pleural effusion with spectral CT image parameters. Thus, clinical findings and spectral CT can provide significant evidence about the nature of pleural effusion[16–18].

Our results should be interpreted in the context of the limitations. First, we only had a small sample of patients in this preliminary study, that could not further evaluate division of pleural effusion. Further studies should be properly classified of causes of pleural effusion based on different type of diseases. Second, in the presence of perfect reference standard, it is not difficult to obtain unbiased estimates of ROC curve to evaluate the classifier accuracy[23]. But in clinical application, there are often imperfect reference standards, for example the small sample size and the confidence level of assigning each instance to its class. This maybe one of the reasons that very high AUC (0.933) was obtained in our study. In the future work, we need to expand the sample size to validate the accuracy of AUC. Third, because only one observer determined the sizes and locations of ROIs, we did not present the interobserver variability. However, the impact would be very limited.

In conclusion, our results demonstrate that dual-energy spectral CT generates effective atomic number, CT values at monochromatic images at 40keV and 100keV from a single scan, and these parameters combined with age and disease history can further improve the diagnostic performance of differentiating benign from malignant pleural effusion.

## Supporting information

S1 Dataset Data for monochromatic images at 40keV and 100keV. (XLS)
